# Anomaly detection method for building energy consumption in multivariate time series based on graph attention mechanism

**DOI:** 10.1371/journal.pone.0286770

**Published:** 2023-06-08

**Authors:** Zhe Zhang, Yuhao Chen, Huixue Wang, Qiming Fu, Jianping Chen, You Lu

**Affiliations:** School of Electronics and Information Engineering, Suzhou University of Science and Technology, Suzhou, China; Vellore Institute of Technology: VIT University, INDIA

## Abstract

A critical issue in intelligent building control is detecting energy consumption anomalies based on intelligent device status data. The building field is plagued by energy consumption anomalies caused by a number of factors, many of which are associated with one another in apparent temporal relationships. For the detection of abnormalities, most traditional detection methods rely solely on a single variable of energy consumption data and its time series changes. Therefore, they are unable to examine the correlation between the multiple characteristic factors that affect energy consumption anomalies and their relationship in time. The outcomes of anomaly detection are one-sided. To address the above problems, this paper proposes an anomaly detection method based on multivariate time series. Firstly, in order to extract the correlation between different feature variables affecting energy consumption, this paper introduces a graph convolutional network to build an anomaly detection framework. Secondly, as different feature variables have different influences on each other, the framework is enhanced by a graph attention mechanism so that time series features with higher influence on energy consumption are given more attention weights, resulting in better anomaly detection of building energy consumption. Finally, the effectiveness of this paper’s method and existing methods for detecting energy consumption anomalies in smart buildings are compared using standard data sets. The experimental results show that the model has better detection accuracy.

## Introduction

Due to the widespread use of intelligent devices, it is now feasible to gather a lot of information on how well they are functioning and evaluate this information to determine whether a building’s energy usage is abnormal. This has become a crucial part of maintaining intelligent building control. Data might be skewed when users fail to turn off their devices after using them or when devices malfunction without their knowledge. The operational status of intelligent building equipment may be accurately sensed by detecting these aberrant data, and the problem of anomaly detection has grown in importance.

The earliest studies where people used statistical methods for anomaly detection included the use of histogram-based statistical methods [1,2] and density-based statistical methods [3,4]. However, these traditional methods are deficient in detecting in the case of complex data and high data dimensionality [5]. An increasingly popular research subject is the issue of energy consumption anomaly detection in the building industry. The literature has undergone a systematic, thorough, and technical study of techniques for anomaly detection of energy usage in buildings [6]. At the same time there are many practical applications in terms of energy consumption, such as the research work related to the estimation of charging demand for electric vehicles presented in the literature [7]. Some researchers have used artificial intelligence algorithms for anomaly identification in light of the ongoing proposal of machine learning-related algorithms. Numerous scholars have learned and mined pertinent features and patterns in the temporal dimension of data using machine learning techniques like neural networks. Pan H et al [8] proposed a deep learning based high-dimensional energy consumption anomaly detection method (HDEC-AD), which uses high-dimensional energy consumption related data to predict customer electricity consumption in real time and perform anomaly detection. lei L et al [9] proposed a building energy consumption data dynamic anomaly detection algorithm (BECMP), which implements dynamic detection of point anomalies and collective anomalies. Shrestha R et al [10] proposed a long short-term neural network (LSTM) based anomaly detection (ADLA-FED) which has a framework for implementing auto encoder in smart grid.A suitable method of detecting building energy anomalies helps to minimize the power consumption of the home while maintaining the comfort of the end user, Atalla S et al [11] proposed a system architecture for improving energy efficiency in residential buildings to promote energy reduction behavior in residential buildings.

Most of the data collected by building smart devices are based on time series [12,13], and time series anomaly detection as one of the problems aims to detect abnormal events from the time series recorded in various regions. A number of researchers have investigated anomaly detection methods based on univariate time series; Chahla et al [14] proposed an anomaly detection method based on long short-term memory and Zhiwei Ji et al [15] proposed an LSTM-based anomaly detection method (LSTMAD), both for identifying anomalous subsequences from univariate time series. Most unsupervised anomaly detection techniques such as clustering, one-class classification, and dimensionality reduction, such as single-class SVM (OCSVM) algorithms, can only detect one type of energy usage anomaly, and in the smart building domain, these univariate time-series anomaly detection methods only analyze energy consumption data without considering other relevant factors that affect energy usage [6]. Among them, Copiaco A et al [16] did the most exhaustive work by analyzing building energy data for anomaly detection. Their innovation in using energy time series images to detect energy consumption anomalies was done by converting 1D energy time series into 2D images, using pre-trained convolutional neural network (CNN) models as feature extractors, and using support vector machines (SVM) to accomplish anomaly classification. The effect of using the hyperparametric variation model as a feature extractor and classifier is also investigated according to the time and resource requirements, and high accuracy is achieved.

However, a significant amount of multivariate time series data is continuously produced by a large number of IoT sensors in numerous practical smart building situations [17–21]. Multivariate time series are formed in smart building energy monitoring systems with multiple sensing measurements, such as room temperature and humidity sensors, water detection sensors, flow meters, etc. As can be seen, univariate time series anomaly detection algorithms are able to detect anomalies in individual metrics but do not represent the overall state of the smart building system well [18,22]. It can be seen that in practical application scenarios, building energy consumption anomalies are not only related to time series but also depend on the correlation between multiple characteristic variables. In recent years, deep learning-enabled anomaly detection (i.e., deep anomaly detection) has emerged as a key direction. Most deep learning-based anomaly detection methods are robust to noise, and such methods can model complex patterns across domains by scaling to higher dimensions. However, they do not have sufficient ability to dig deeper into potential correlations from multivariate time series, which leads to lower detection accuracy and recall. Many researchers have used machine learning methods such as neural networks to learn and mine relevant features and patterns in the temporal dimension of data. Su Y et al [23] proposed OmniAnomaly, a stochastic recurrent neural network for multivariate time series anomaly detection that works well robustly for various devices. Li D et al [24] proposed Multivariate Anomaly Detection with GAN (MAD-GAN) framework considers the entire variable set concurrently to capture the latent interactions amongst the variables. Park D et al [25] proposed a long short-term memory-based variational autoencoder (LSTM-VAE) that fuses signals and reconstructs their expected distribution by introducing a progress-based varying prior. However, these methods do not easily detect small anomalies in the data, while paying less attention to the analysis and mining of correlations between different variable dimensions [26], do not explicitly address the problem of capturing the correlation of multivariate time series, and there is still room for improvement in properly mining the relationships between different time series.

In real-world application settings, energy consumption anomalies are determined by the correlation between numerous features and the time series. In this scenario, just taking into account single time series to identify anomalies across the board is biased; instead, it is best to start by taking into account multiple time series feature correlations. In this study, the key research question is how to mix time series and multivariate characteristics in the anomaly detection problem. As a result, the method for multivariate time series anomaly identification proposed in this research concentrates on mining the correlation between various multiple time series and on the detection of anomalies in the overall state. Firstly, a graph convolutional network is introduced to model the topological relationships between feature variables by taking multiple time series variables as input and extracting feature correlations between multiple feature variables. Secondly, a graph attention mechanism is introduced to improve the detection framework in order to prioritize certain variables with considerable potential interrelationships between time series. Anomaly detection models are constructed by extracting correlations between individual time series of multivariate features and weighting the sum of features with the graph attention mechanism to model complex time series data. This method is tested with existing anomaly detection methods using an open standard dataset, and the experimental findings demonstrate that the model has superior detection accuracy than existing anomaly detection methods.

The contributions of our paper are summarized as follows:

1. we propose a multivariate time series anomaly detection method based on multivariate time series, considering multiple factors that lead to anomalies in building energy consumption. A graph convolutional network is used to construct the anomaly detection framework and extract feature correlations between multiple feature variables.
2. This paper introduces a graph attention mechanism to better achieve anomaly detection of building energy consumption by calculating the attention coefficients between different time series features and assigning greater attention weights to the time series features with greater influence on energy consumption.
3. The method shown in this paper is contrasted with other anomaly detection techniques using an open standard dataset. The findings show that the anomaly detection method has a higher detection precision.
4. The structure of this paper is as follows: Section 2 provides preparatory work on multivariate time series anomaly identification. The design and application of the approach are given in Section 3. Experiments and analysis are done in Section 4. A summary of the entire paper is provided at the end.

### Preparatory work

#### Problem description

**Fig 1:**
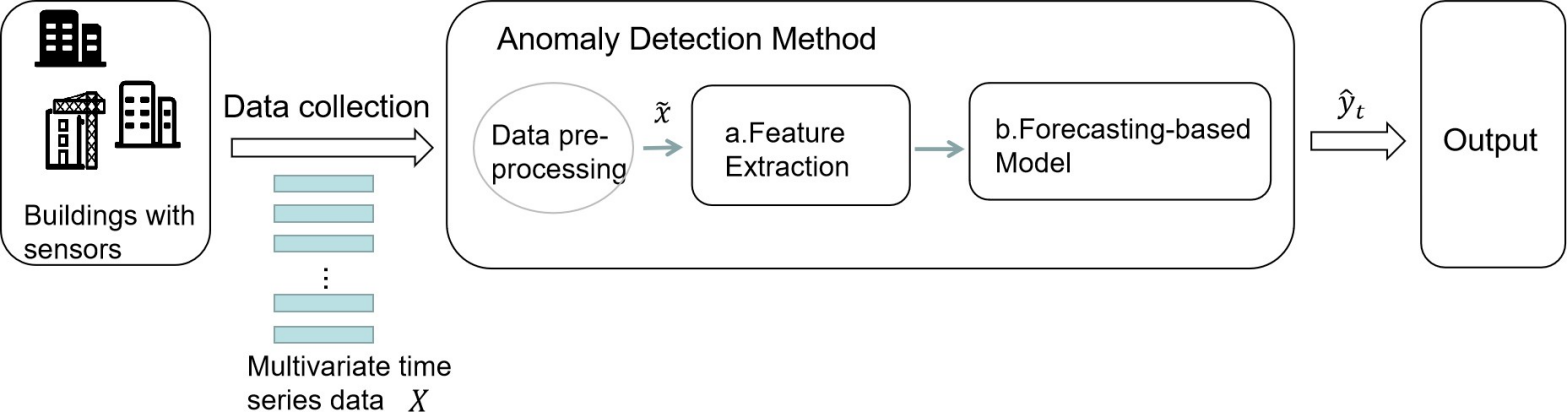
A Schematic diagram of the overall process.

The goal of anomaly detection is data driven to find anomalies in all samples. The problem can be defined as follows:

Assume there are several buildings in a residential area, symbolized by the symbol

$$P=\{A_{1},A_{2}\ldots \ldots A_{m}\},$$ (1)

where P denotes the region’s collection of buildings and A_i_ denotes the number of building i. There are m buildings and each one has k sensors that are used to collect data. The data are time series data, and the characteristic elements that correspond to the data will have an effect on how much energy each building uses. The multivariate time series data are recorded as $x_{j}^{i}$, and the data gathered by sensor j at time t in building I are recorded as

$$X=\left[\begin{array}{cccc} x_{1}^{1} & x_{1}^{2} & \ldots & x_{1}^{k}\\ x_{2}^{1} & x_{2}^{2} & \ldots & x_{2}^{k}\\ \ldots & \ldots & \ldots & \ldots \\ x_{m}^{1} & x_{m}^{2} & \ldots & x_{m}^{k} \end{array}\right]\in R^{m*k},$$ (2)

where $L_{i}={\left[x_{1}^{i},x_{2}^{i},\ldots \ldots x_{m}^{i}\right]}^{T}$, $L_{i}\in R^{1*m}$, L_i_ represents the multivariate time series data collected for the characteristic factors that have an impact on the abnormal energy consumption of the buildings. Each column in the formula represents a measured variable, and each row represents a collected sample. Therefore, this problem can be transformed into a training set based on the historical data collected from buildings with sensors as the input data X. The data is preprocessed to obtain data $\tilde{x}$. After the anomaly detection model is trained, the energy consumption data with anomaly labels are used as the test set data to detect them, and the final output is y. The overall flow is shown in Fig 1.

### Related definitions

A time series is a collection of observations that are continuously measured over time to track changes. Observations are typically made at evenly spaced time intervals

$$T=(t_{0}^{d},t_{1}^{d},\ldots \ldots t_{t}^{d}),d\in N_{+},t\in N,$$ (3)

where d indicates how dimensional the time series is. There are two different types of time series based on the characteristics of the observations, and time series often assume that the time intervals are equal. On the one hand, a time series is a univariate time series if its observations are scalar values. On the other hand, the time series is a multivariate time series if the observations are multidimensional vectors.

Definition 1 **Multivariate time series:**

This paper focuses on multivariate time series where each variable depends not only on its past values but also on other variables. For example, the multivariate time series data in this paper is denoted as:

$$X=\left[\begin{array}{cccc} x_{1}^{1} & x_{1}^{2} & \ldots & x_{1}^{k}\\ x_{2}^{1} & x_{2}^{2} & \ldots & x_{2}^{k}\\ \ldots & \ldots & \ldots & \ldots \\ x_{m}^{1} & x_{m}^{2} & \ldots & x_{m}^{k} \end{array}\right]\in R^{m*k},$$ (4)

where L_i_∈R^1*m^, for example, the electricity consumption data in kWh in the building is the time series data, denoted as:

$$L_{1}={\left[x_{1}^{1},x_{2}^{2},\ldots \ldots x_{m}^{1}\right]}^{T}$$ (5)

the temperature in the area where the building is located is also an important factor affecting the detection of energy anomalies in the building, denoted as:

$$L_{2}={\left[x_{1}^{2},x_{2}^{2},\ldots \ldots x_{m}^{2}\right]}^{T}$$ (6)

Definition 2 **Exception:**

**Fig 2:**
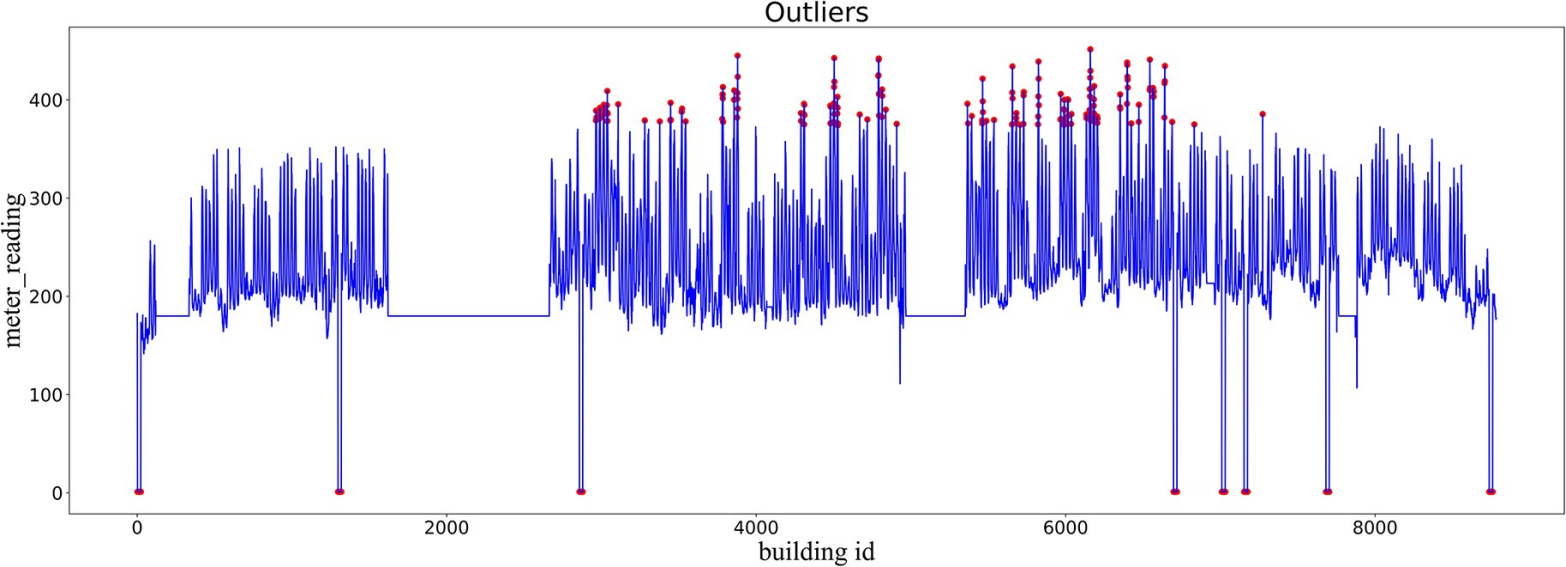
Outlier situation.

Users forget to turn off their devices after use or some devices fail without knowing it, which can lead to deviations in the data, and these deviated data are called anomalous data. Anomalous data is distinctly different from the overall distribution of data. The set of anomalous data constitutes a very small part of the data set. The horizontal coordinate is the building id, and the vertical coordinate is the energy consumption information for each building id, as illustrated in Fig 2 anomalies. The figure shows red for aberrant data and blue for normal data. The data distribution that deviates significantly from the overall distribution of the data is called an anomaly.

### Related Theories

#### Graph convolutional network

**Fig 3:**
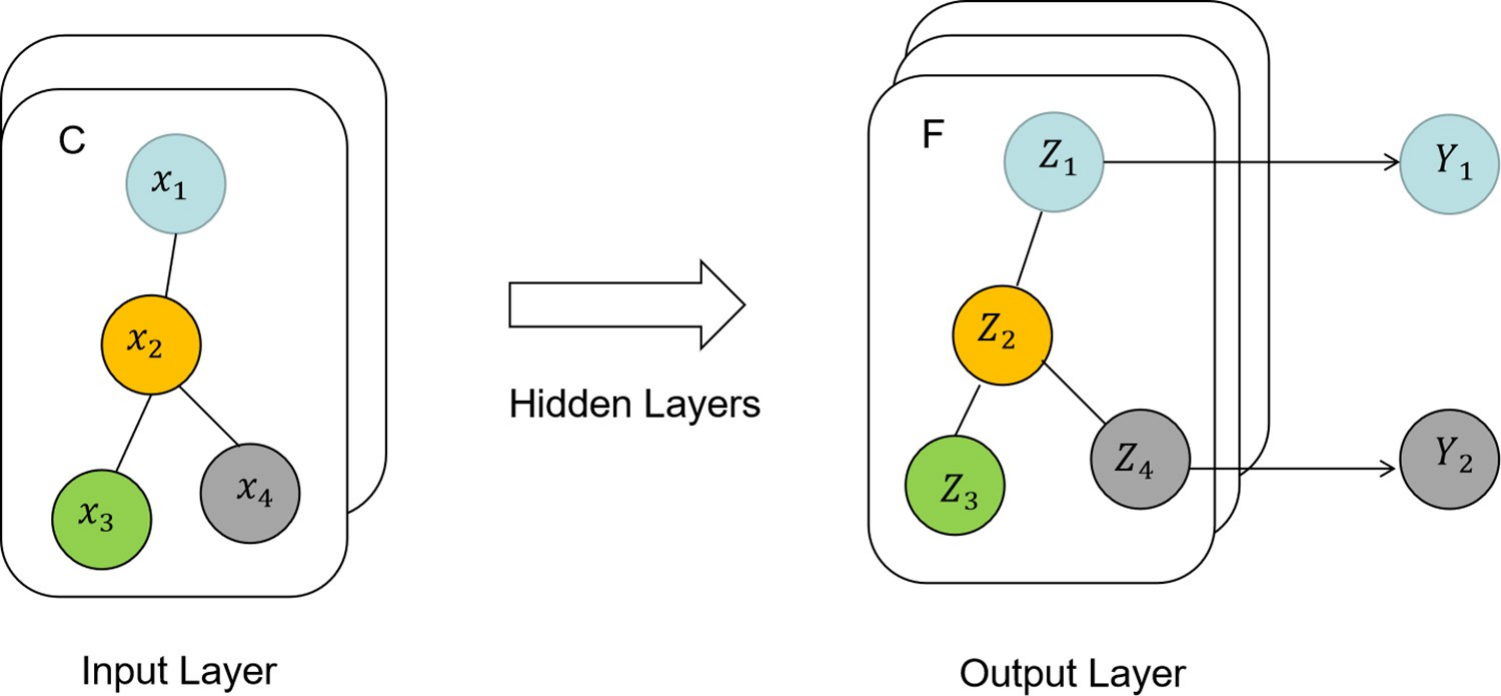
Schematic diagram of the graphical convolutional network.

**Fig 4:**
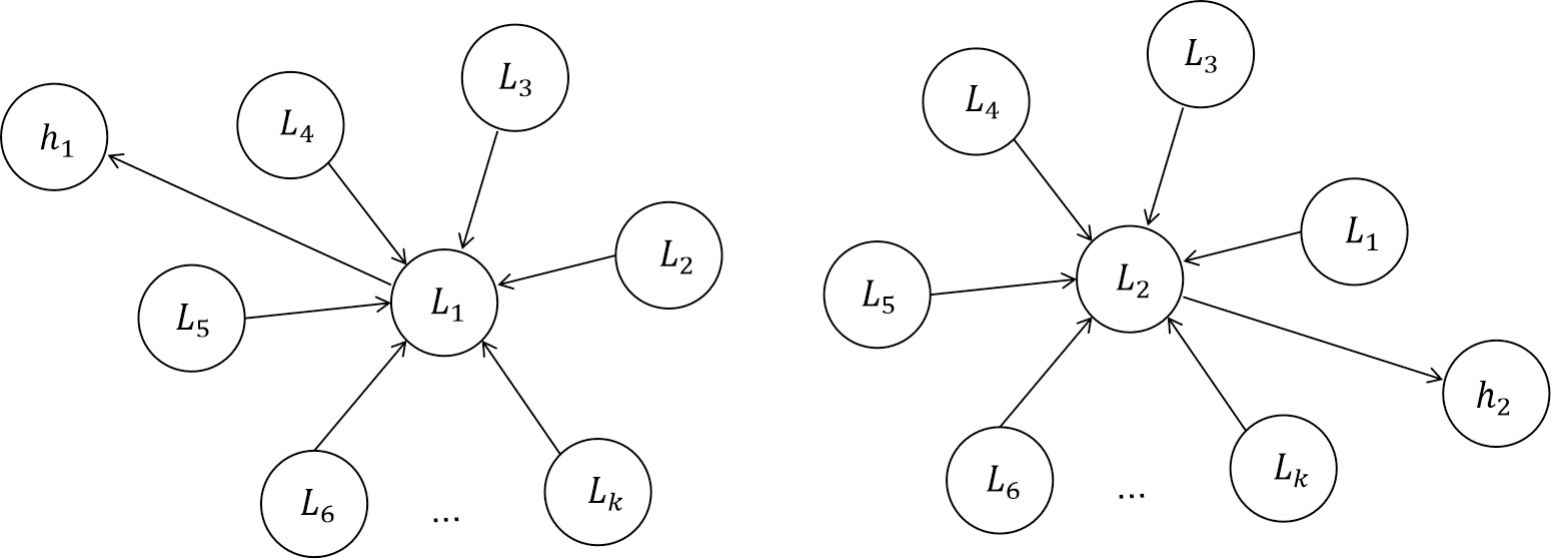
Feature node diagram structure.

The Graph Convolutional Network (GCN), first described by Kipf and Welling [27], is one of the most established and successful models of graph neural networks. It introduces symmetric normalization and self-loop aggregating techniques based on neural networks. A graph convolutional network [28] is schematically represented in Fig 3, with C input channels and F feature mappings that are labels for the relevant nodes. The graph convolution layer uses the information of edges to aggregate node information, creating a new node representation, and propagates neighborhood associations between layers by aggregating nearby nodes. As a result, the multivariate time series X is viewed in this paper as a complete graph, where each node denotes a specific feature and each edge denotes the relationship between two corresponding features. The graph structure is shown in Fig 4. This paper uses a graph convolutional network to extract correlations between different feature variables.

The multivariate time series data (data collected by sensors carrying a large amount of environmental information) affecting the building energy consumption anomalies are constructed as a graph model, and the different types of environmental information data collected are used as nodes in the graph model, and the correlation between different dimensions of data is represented by node-to-node edges. This graph-based approach is more suitable for using graph convolutional neural networks. The graph convolution layer aggregates neighboring nodes to enable the transfer of neighborhood relationships and propagates between layers, and uses the information of edges to aggregate node information to generate a new node representation. GCN provides a method to model the relationship between sensors by representing the correlation between nodes to obtain an embedded representation between nodes.

#### Graph attention network

In recent years, graph neural networks have become one of the most prominent model algorithms in the field of deep learning. It focuses on data in the form of graphs and investigates the links between nodes as well as the relationships between edges. Because of its inherent properties, it is ideal for depicting the interactions between different nodes. Graph neural networks, on the other hand, require a specific graph structure in the data and are not ideal for dealing with anomaly detection problems in time-series data.

Previous multivariate time series anomaly detection tends to ignore the correlation between features and idiosyncratic evidence, for multivariate time series, the correlation between different feature variables is important. Different variables have different importance, i.e., different weights for the points to be measured. The information in the feature dimension is critical for anomaly detection, but previous models did not pay attention to this dimension. In the process of applying GCN we found that there are still shortcomings, mainly that the GCN method captures features with fixed edge weights when neighboring nodes are featured, i.e., the degree of influence of different dimensions of environmental information is considered uniform. However, in practice different environmental factors have different degrees of influence on energy consumption, so the edge weights should not be fixed. This problem needs to be taken into account by introducing the graph attention mechanism (GAT), which can highlight the contribution of different neighbor nodes to the central node in the aggregated graph convolution information and can assign greater attention weights to the factors with greater influence on energy consumption, which is conducive to better anomaly detection. To make full use of the information about the importance of each variable in the feature dimension, we apply the Graph Attention Network(GAT) to the feature dimension in this paper, which is why we choose GAT.

**Fig 5:**
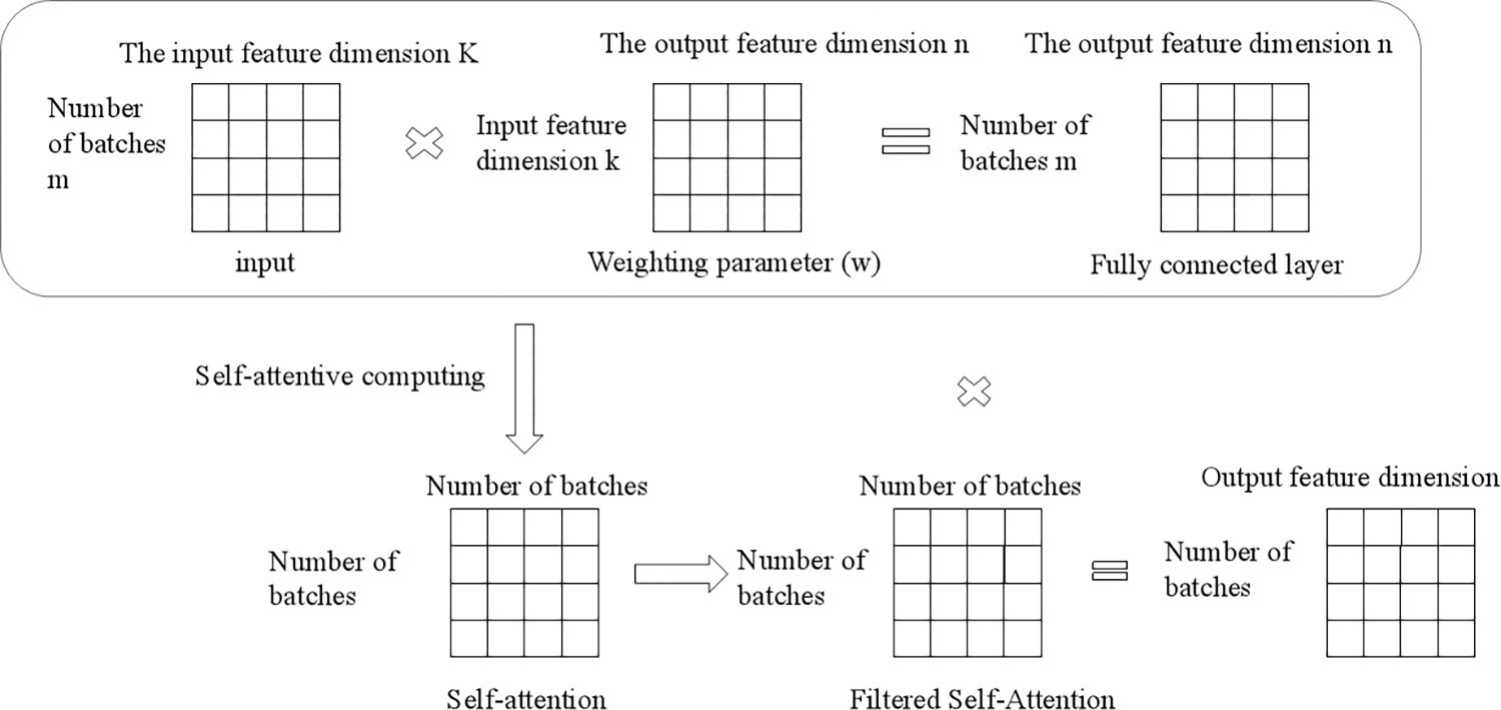
Graphical attention network.

The core of GAT first creates a graph structure with multiple feature variable nodes, learns the importance of each feature element from the sequence to obtain the weight corresponding to each feature node, and merges the elements in accordance with the importance. The weight parameter is a coefficient that the graph attention mechanism assigns to the elements. The GCN layer gains a hidden self-attention (self-attention) from GAT. With the self-attention layer overlaid, different nodes in the neighborhood are given varying degrees of priority during the convolution process while dealing with neighborhoods of various sizes. This study employs GAT to compute the correlation between nodes in this model in order to determine the potential relationship between variables, and its structure is shown in Fig 5. The GAT layer is used to calculate the correlation between nodes, and the output is *h*_*i*_, i∈(0, k).

### Method design and implementation

#### Model framework

In the building sector, there are frequently many sensors collecting data and complicated topological links between them. The entire system may be thought of as a graph structure, with each sensor acting as a particular node inside the graph.

**Fig 6:**
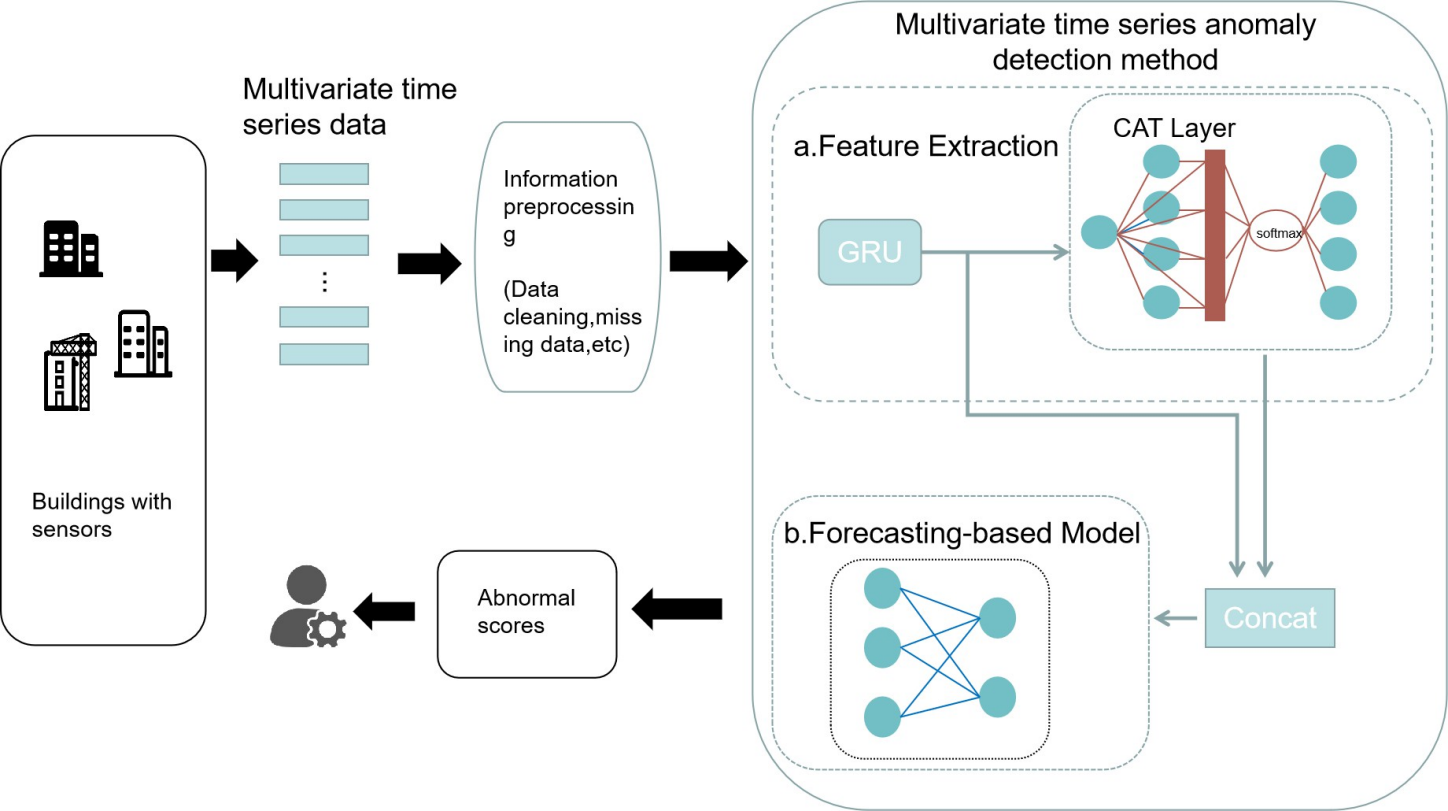
Model framework.

The proposed method introduces the graph attention mechanism in GCN and combines it with LSTM networks. Among them, the graph attention model (GAT) replaces the fixed normalization operation in the graph convolution with the attention mechanism. The relationship between multivariate time series is fully explored, and the dependence of time series on feature dimensions is explored to better detect building energy consumption anomalies. The overall model framework is shown in Fig 6:

The method consists of the following modules in sequence:

1. Data cleaning and dimensionality reduction operations are carried out on the original dataset in data pre-processing due to the enormous amount of data and complex data structure of the dataset.
2. The processed data are first passed through the GRU layer while maintaining the feature dimension. The series is then remodeled by introducing a convolutional neural network with a graph attention mechanism, which is used to extract the correlation between each time series of multivariate features by weighted summation of neighboring node features.
3. The long-term and short-term memory neural network layers are fed jointly to the convolutional neural network’s output representation with the graph attention mechanism added, and the input to the prediction-based model is obtained from the prediction error.

### Figure attention layer

The method used to gather and accumulate the feature representations of adjacent nodes at a distance of one is the main distinction between the graph attention layer and the GCN. One graph convolution process in GCN entails summing the normalized node feature values.

$$h_{i}^{\left(l+1\right)}=\sigma \left(\sum_{j\in N(i)}\frac{1}{c_{\mathrm{ij}}}W^{\left(i\right)}h_{j}^{\left(l\right)}\right)$$ (7)

where *N*(*i*) is the set of nodes neighboring node i at distance 1. An edge connecting node i to itself is usually added so that i itself is included in *N*(*i*). $c_{\mathrm{ij}}=\sqrt{\left|N(i)\right|}\sqrt{\left|N(j)\right|}$ is a normalization constant based on the graph structure; *σ* is an activation function (GCN uses ReLU); *W*^(*i*)^ is a weight matrix for node feature transformation, shared by all nodes.

The graph attention model (GAT) replaces the fixed normalization operations in graph convolution with an attention mechanism [29] and is one of the most popular graph neural networks. The graph is represented as G = (V, E), where V denotes the set of nodes and E is the set of edges. The number of nodes is denoted by K, and V_ij_ = (i,j) is defined to denote an edge from i to j. The time series data collected by different sensors in the system are interdependent, where the dependencies between different sensors can be modeled as graph structured data.

The multivariate time series are viewed as a complete graph, where each node is a specific feature and each edge denotes the relationship between two related features, in order to mine the feature relationships between the multivariate time series. GAT is used in this manner to systematically record the dependencies between surrounding nodes.

Each node *L*_*i*_ is represented by m $x_{j}^{i}=\{x_{j,t}^{i}|t\in (0,n)\}$’s, and there are k nodes in total, where n is the total number of timestamps, k is the total number of multivariate features, and m represents the number of buildings, i.e., the total number of time series collected by each feature node. Since the influence of different neighbors on the central node is different, GAT is introduced to automatically learn this weight parameter by paying attention, thus improving the characterization ability. The input of GAT is the set of node feature vectors, denoted as:

$$L=\{L_{1},\;L_{2},\ldots \ldots ,\;L_{k}\},\;L_{i}\epsilon R^{p},$$ (8)

where P denotes the dimension of each node vector. The output of GAT is a new set of node feature vectors, denoted as:

$$L^{\prime }=\{L_{1}^{\prime },\;L_{2}^{\prime },\ldots \ldots {,L}_{k}^{\prime }\},L_{i}^{\prime }\epsilon R^{p^{\prime }},$$ (9)

where *p*′ is the dimension of each output node vector.

To obtain a sufficient expression, the output of each node can be expressed as Eq (10), which defines how to do an update of the lth layer node features to get the l plus 1 layer node features Eq (10) does a linear transformation of the lth layer node embedding $L_{i}^{\left(l\right)}$, and *W*^(*l*)^ is the trainable parameter of this transformation. The original attention score between each node is computed by Eq (11), which first splices two node embeddings, followed by a dot product of the spliced embeddings and a learnable weight vector; finally, a LeakyReLU activation function is applied. This form of attention mechanism is often referred to as "additive attention" and is distinct from the "dot product" attention in Transformer.


$$L_{i}^{\left(l\right)}=W^{\left(l\right)}h_{i}^{\left(l\right)}$$ (10)

eij(l)=LeakyReLU(a−(l)T(Li(l)||Lj(l))), (11)

Subsequently, Eq (12) applies a softmax operation to the original attention scores obtained for all incoming edges of a node to obtain the attention weights. Eq (13) resembles the node feature update rule of GCN and does an attention-based weighted summation of the features of all neighboring nodes.


$$\alpha_{\mathrm{ij}}^{\left(l\right)}=\frac{\exp(e_{ij}^{\left(l\right)})}{\sum_{k\in N(i)}\exp(e_{ik}^{\left(l\right)})}$$ (12)

$$L_{i}^{\left(l+1\right)}=\sigma (\sum_{j\in N(i)}\alpha_{ij}^{\left(l\right)}L_{j}^{\left(l\right)})$$ (13)

**Fig 7:**
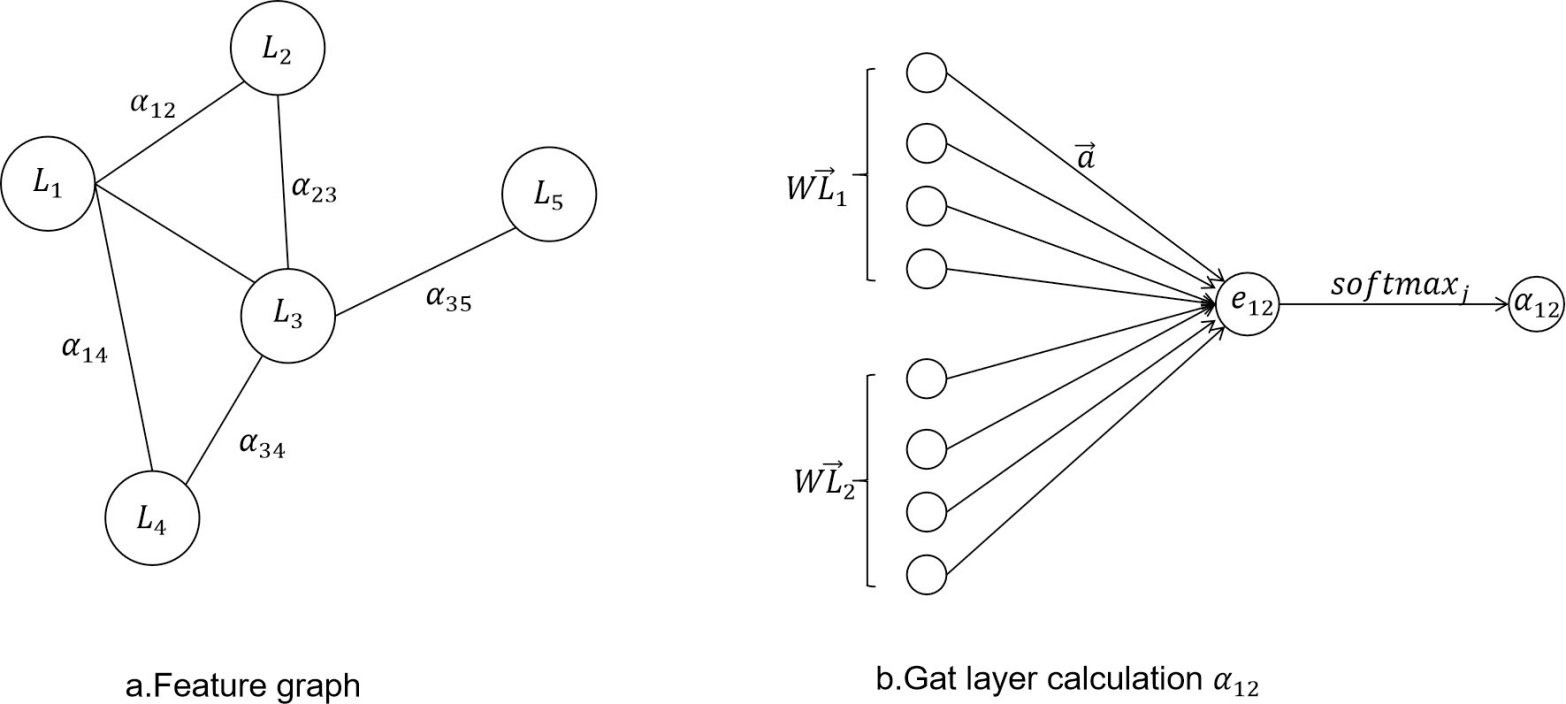
Example. a introduces the Feature graph part, b introduces Gat layer calculation α_12_ part.

In the multivariate time series anomaly detection task, the multivariate time series is considered a complete graph, as shown in Fig 7(A). Five feature relationships are selected as an example, and the relationship between the multivariate time series collected by the feature factors is shown in Figure a. *α*_*ij*_ represents the weight value between feature factors *L*_*i*_ and L_j_. Use GAT to calculate the weight values between each feature node; take the feature factors *L*_1_ and *L*_2_ as an example, as shown in Fig 7(B). GAT linearly transforms the two nodes separately, and the original attention coefficient e_12_ between each node is calculated by Eqs (10) and (11), then the attention coefficient e_12_ is assigned to the nodes in the graph using the mapping function, and in order to compare the attention coefficients between different nodes, the attention weight b is obtained by normalizing using the softmax function. $W{\vec{L}}_{i}$ represents the use of a shared W vector learned by the model itself to do the dimensional transformation on the original feature vector, i and j are transformed after splicing, and then use a full connection as the similarity calculation function, the activation function is LeakyRelu, at this time after the full connection output All the fully connected values are then normalized by softmax to obtain the final ij node weights. The information transfer between nodes is achieved by the properties of edges, and each update is aggregated to its neighboring nodes. The interrelationships between time series are learned by GAT.

### Feature prediction layer

The model framework includes a prediction-based model to predict the value of the next timestamp, and the parameters of the model are updated during the training process. Because it is multivariate, this paper uses a sigmoid function that can select multiple variables, and finally, the feature-based prediction result $y_{t}^{\prime }$ is linearly transformed with the original data *x*_*t*_ as the input to the LSTM network, and the output result is ${\hat{y}}_{t}$. Three fully connected layers with hidden dimension d2 are stacked after the LSTM network, and the root mean square error (RMSE) is chosen for the loss function of the prediction model as follows:

$$\mathrm{Los}s_{f}=\sqrt{\sum_{i=1}^{k}({\hat{y}}_{t,i}-x_{t,i}{)}^{2},}$$ (14)

where ${\hat{y}}_{t,i}$ denotes the predicted value of the ith feature passing through the prediction model at moment t, and x_t,i_ denotes the true value of the ith feature at moment t. The test set is fed into the trained prediction model, and the root mean square error between the predicted and true values at each moment is denoted as {l_1_, l_2_,……l_n_}∈R^N^.

For each timestamp, the inference score s is calculated for each feature using the prediction value $\left\{{\hat{x}}_{i}|i=1,2,\ldots \ldots k\right\}$ calculated based on the prediction model, and the sum of all features is taken as the final inference score. If its corresponding inference score is greater than the threshold, we consider this timestamp as an exception, and we use the peak over threshold (POT) to automatically select the threshold. Specifically, the inference score can be calculated by:

$$score=\sum_{i=1}^{k}s=\sum_{i=1}^{k}\sqrt{\left({\hat{x}}_{i}-x_{i}\right)}$$ (15)

### Anomaly detection

First, the original data X∈R^m×k^ is input into the GRU layer, keeping the feature dimension constant, to obtain U∈R^m×k^. u_t_ is the output of GRU at moment t. Subsequently, the obtained output is subjected to a one-dimensional convolution operation on the row vectors of U_t_ by a convolution kernel to obtain the deep convolutional features of each feature variable in the time dimension. All the variables go through the hidden state of the K feature variables to obtain the matrix A‘ϵR^m×k^’, and the activation function is selected as ReLU. Consequently, a convolutional neural network with a graph attention mechanism is input to extract correlations between individual time series of multivariate features using a weighted summation of neighboring node features with the graph attention mechanism to re-model the series. Finally the joint and data are input to the prediction-based model, and the threshold is automatically selected according to the POT to determine the anomaly score and feedback to the system manager.

The algorithm flow is described in Algorithm 1 and Algorithm 2.

**Algorithm 1**. Training GCN

**Input**: Given dataset X, Learning rate *η*,Number of iterations t

**Output**: Matrix O

1. Initialize *W*^(0)^,*b*^(0)^,*W*^(1)^,*b*^(1)^.

2. **for** iteration = 1:t

3.  Layer_1_output = X *L *W^(0)^−*b*^(0)^;

4.  Layer_2_output = X*Layer_1_output*W^(1)^−*b*^(1)^;

5.  Loss = Cross-entropy;

6. Update W and b using batch gradient decent ∇w(Loss);

7. **end**

8. **return O**

**Algorithm 2.** Anomaly detection method based on graph attention mechanism

**Input**: training data *X*_*train*_, testing data *X*_*text*_

**Output**: anomaly or no anomaly

**At training model stage**:

1. Initialize the parameters of the model.

2. **While** not converged do

3.  training data *X*_*train*_;

4.  **for** each data in the training data do

5.   Training GCN;

6.   Calculate the correlation between different feature dimensions;

7.   Update variable dimension representation *v*_*i*_;

8.   Calculate forecast results;

9.   Calculate the prediction error of the model;

10  **end**

11.  Calculate the total loss of the model and update the parameters according to stochastic gradient descent.

12. **end**

**At anomaly detection stage**:

13. Calculate anomaly score.

14. Calculate average anomaly score for each point of time series corresponding to the testing data *X*_*text*_.

15. if (score > threshold):

16.  return anomaly;

17. else:

18.  return no anomaly.

## Experiment and analysis

### Experimental setup

#### Dataset

This paper uses the dataset used in the Large Energy Predictor III competition2 conducted on the Kaggle platform [30], a standard dataset for large building energy anomaly detection. The dataset includes 1,636 one-year hourly meter readings for non-residential buildings collected from 16 different locations around the world [31]. In addition, it contains building metadata, such as square_feet, year_build, and floor_count, to describe the structure of the building (specified by building_id). It is also accompanied by various weather parameters to help better model the energy use of the building. The measurements in this dataset were taken from four different meter types (electric, cold water, steam, and hot water). For anomaly detection, hourly meter reading data was taken from 1,413 meters covering 16 different building types, such as offices, and monitored for one year. Over the course of a year, each data point in the meter readings was annotated for both abnormal (1) and normal (0) use. Each building has one or more meters measuring electrical, heating and cooling water, steam and solar, and water and irrigation throughout the building. This paper describes the data collection, cleaning of time series meter data, building of metadata, and supplemental weather data. This dataset can be used for further predictive benchmarking and prototyping as well as anomaly detection, energy analysis, and building type classification.

### Data pre-processing

(i) Missing value handling

Missing data is a situation in which data are incomplete for some reason during data collection, transmission, and processing. The presence of missing values can cause problems in the statistics of the data, and certain data analysis models cannot directly deal with datasets with missing values [30]. If further statistics and analysis of this data are to be performed, the first step is to deal with the missing values.

(ii) Eliminate irrelevant features

It is unknown which features are effective for a particular learning algorithm. Therefore, the relevant features that are beneficial for the learning algorithm need to be selected from all the features. Moreover, in practical applications, the problem of dimensional catastrophe often arises. If only some of all the features are selected to build the model, the running time of the learning algorithm can be greatly reduced and the interpretability of the model can be increased.

(iii) Data normalization

To improve the robustness of the model, data normalization and cleaning are performed for each individual time series. Data normalization is applied to both the training and test sets.

$$\overset{˜}{x}=\frac{x-\min(X_{train})}{\max(X_{train})-\min(X_{train})}$$ (16)

where max(X_train_) and max(X_train_) are the maximum and minimum values of the training set, respectively.

**Fig 8:**
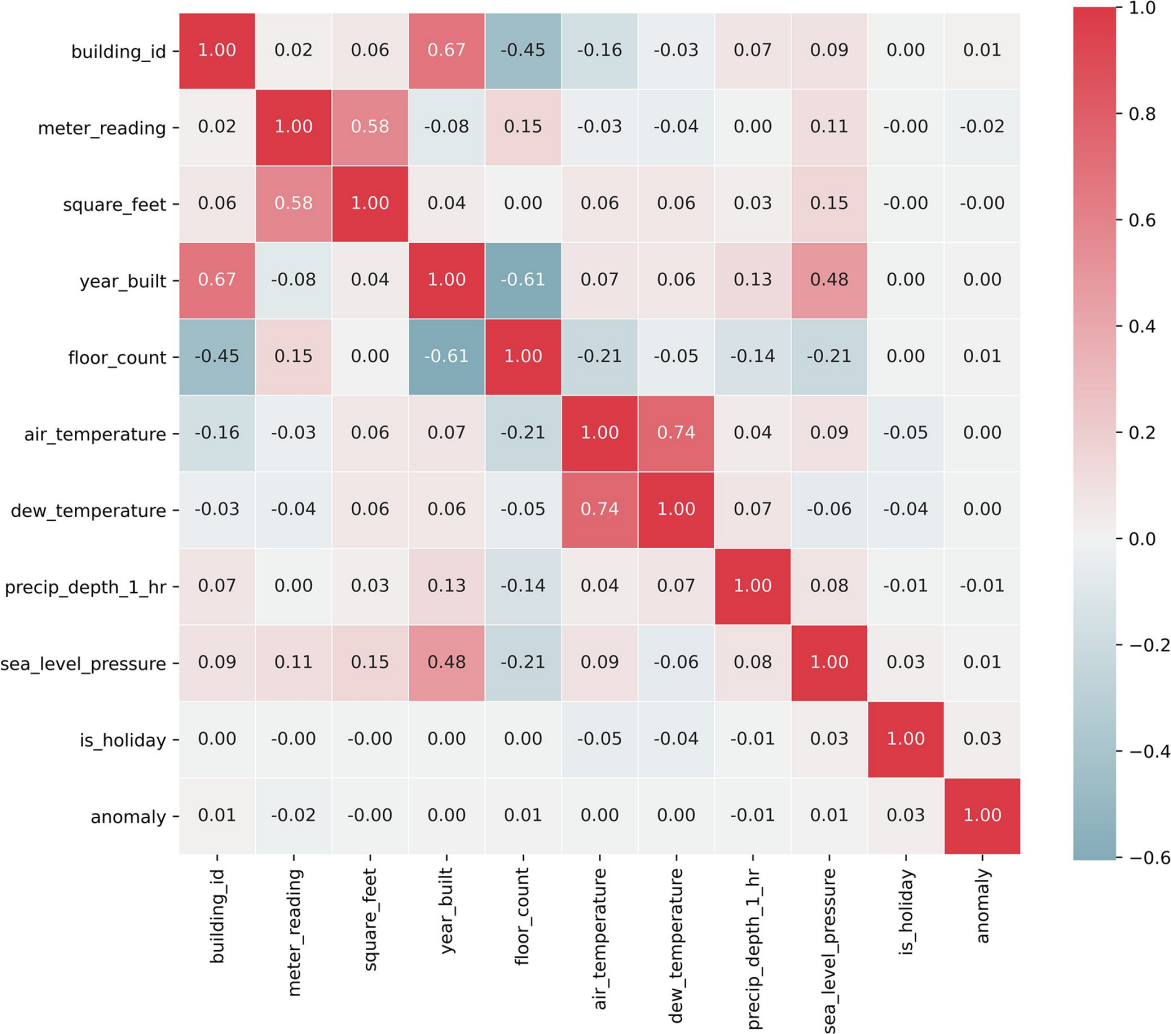
Characteristic heat map.

Data missing value processing, elimination of irrelevant features, and data normalization operations were performed on the dataset in turn. For the processing of data missing values, the simple and easy-to-operate deletion method was chosen in this paper, because the dataset was large enough that deleting certain samples did not cause loss of the original information of the data. The feature selection of the original dataset was performed using the correlation coefficient function, and 11 features that may affect the detection of building energy consumption anomalies were selected. The heat map is shown in Fig 8, and the dimensions of the processed dataset are shown in Table 1.

**Table 1:** Data set dimensions.

Datasets	Building Energy Standards Dataset
Number of sequences	11
Training set size	478880
Testing set size	569695
Test_label	569695
Anomaly Rate(%)	34.6

#### Evaluation metrics

The performance of the model is expressed using precision, recall, and F1-scores. The performance measures are predicted precision and recall, and, respectively, their definitions are shown in (17)–(19). In the definitions, TP (true positive) indicates the number of abnormalities that are truly (or correctly) detected as abnormal; FP (false positive) indicates the number of normal data that are incorrectly (or incorrectly) detected as abnormal; TN (true negative) indicates the number of normal data that are truly detected as normal; and FN (false negative) indicates the number of abnormalities that are incorrectly detected as normal.


$$\mathrm{precision}=\frac{TP}{\left(\mathrm{TP}+FP\right)}$$ (17)

$$\mathrm{recall}=\frac{TP}{\left(\mathrm{TP}+FN\right)}$$ (18)

$$F_{1}-score=2\times \frac{\mathrm{precision}\times \mathrm{recall}}{\mathrm{precision}+\mathrm{recall}}$$ (19)

#### Comparison method

In this paper, classical methods for anomaly detection and current advanced algorithmic models are selected for comparison, as shown in Table 2. There are statistical-based methods as well as machine-learning-based methods for benchmarking anomaly detection on the dataset.

**Table 2:** Comparison methods.

Categories	Methods	Thoughts
Traditional methods	Cluster-based local outlier factor (CBLOF) [32]	A local outlier detection method.
	Feature Bagging [33]	An integration method.
	Histogram-based outlier detection (HBOS) [34]	semi-supervised learning for anomaly detection.
	Isolated Forest [35]	A subspace-based approach.
	K-Nearest Neighbor (KNN) [36,37]	A method based on distance calculation.
	Local Outlier Factor (LOF) [38]	An unsupervised anomaly detection method
	Minimum covariance determinant (MCD) [39]	A statistical based anomaly detection method.
	Single Class SVM (OCSVM) [6]	A semi-supervised anomaly detection method.
Advanced methods	OmniAnomaly [23]	Stochastic recurrent neural network anomaly detection method.
	MAD-GAN [24]	Unsupervised multivariate anomaly detection method for generating adversarial networks.
	LSTM-VAE [25]	A long short-term memory-based variational self-encoder.
	GDN [40]	Graph Deviation Network(GDN) is an unsupervised anomaly detection method.

The same sliding window size, n = 100, is used for all models. The hidden dimension size based on the prediction model is empirically set to 300. The Adam optimizer is used to train the model for 100 epochs with an initial learning rate of 0.001. The effectiveness of the method is demonstrated by comparing it with these anomaly detection methods.

### Performance comparison

Table 3 summarizes the comparison of the method in this paper with classical methods of anomaly detection and current advanced algorithmic models in terms of F1-score, accuracy, and recall on the same data set. The method in this paper shows excellent generalization ability and obtains the best F1-score on this dataset, and the F1-score is better than the optimal baseline GDN, which can learn as many time series features from different edge devices as possible, thus improving the robustness of the model. In the classic method model, the K-nearest neighbor and the minimum covariance determinant yield the highest precision, i.e., 0.902 and 0.901, respectively. the method in this paper is 0.822 in terms of precision, which is slightly lower than the above two models, but much higher than the above two models in terms of recall and F-score. Compared with most methods, classic methods (e.g., CBLOF, Feature Bagging, etc.) perform poorly, with lower F1-scores and recall rates, although the precision is generally higher. It can be seen that these traditional methods are deficient in detecting the effect in the case of complex data and high data dimensionality. In addition, current advanced methods (e.g., OmniAnomaly, MAD-GAN, LSTM-VAE, GDN) improve F1-scores and recall rates over classical methods while maintaining high accuracy, and it is clear that learning and mining relevant features and patterns in the temporal dimension of the data using machine learning methods such as neural networks is beneficial for improving anomaly detection methods accuracy. Recent GDNs have achieved better performance than other baselines. However, GDN is not good at obtaining temporal features from time series.

**Table 3:** Comparison of F1-score, Precision, and Recall of anomaly detection models.

Model	F1-score	Precision	Recall
Cluster-based Local Outlier Factor (CBLOF)	0.425	0.900	0.277
Feature Bagging	0.424	0.899	0.279
K-Nearest Neighbors (KNN)	0.431	0.902	0.284
Histogram-base Outlier Detection (HBOS)	0.397	0.896	0.258
Isolation Forest	0.413	0.895	0.270
One-class SVM (OCSVM)	0.421	0.899	0.276
Local Outlier Factor (LOF)	0.426	0.900	0.281
Minimum Covariance Determinant (MCD)	0.422	0.901	0.276
OmniAnomaly	0.843	0.942	0.978
MAD-GAN	0.813	0.805	0.821
LSTM-VAE	0.729	0.856	0.643
GDN	0.852	0.748	0.989
**Ours**	**0.877**	**0.822**	**0.940**

GDN is an unsupervised anomaly detection method that uses the graph attention mechanism to learn structure in multivariate time series and explains the detected anomalies by attention weights. The experimental results demonstrate that GDN has a better advantage of using the graph attention mechanism to learn correlations between sensors, and the experimental results F1-score of 0.852 are higher than those of univariate time series-based anomaly detection methods, and for multivariate time series-based anomaly detection methods (OmniAnomaly, MAD-GAN, LSTM-VAE), GDN outperformed the experimental results of the above methods, indicating that the graph attention mechanism plays an important role in the anomaly detection process. However, the method in this paper outperforms GDN in F1-score and Precision, which indicates that the combined performance of GCN and GAT is better than that of using GAT only. And the GDN method is not good at obtaining temporal features from time series.

Among them, LOF, MCD, and CBLOF these anomaly detection methods can only identify energy consumption outliers based on the analyzed energy consumption level, but cannot detect other anomalies, which has certain limitations, so the experimental results are not satisfactory. Feature Bagging is one of the common methods used in integration methods, where multiple datasets are obtained by resampling the features of a sample, and then a set of models are used to train on these datasets. Isolation Forest is advantageous in handling large datasets because of the time efficiency of this algorithm, but it is not applicable to ultra-high dimensional data. The advantages of the KNN algorithm for detecting outliers in time-series data are short training time, no assumptions on the data, and high accuracy; the disadvantages are high computational effort, low accuracy for rare categories, and poor interpretability. HBOS is a combination of univariate methods that is faster to compute and friendly to large data sets, but cannot model the dependencies between features. So the above methods do not show good performance in dealing with multivariate time series anomaly detection problems.

**Fig 9:**
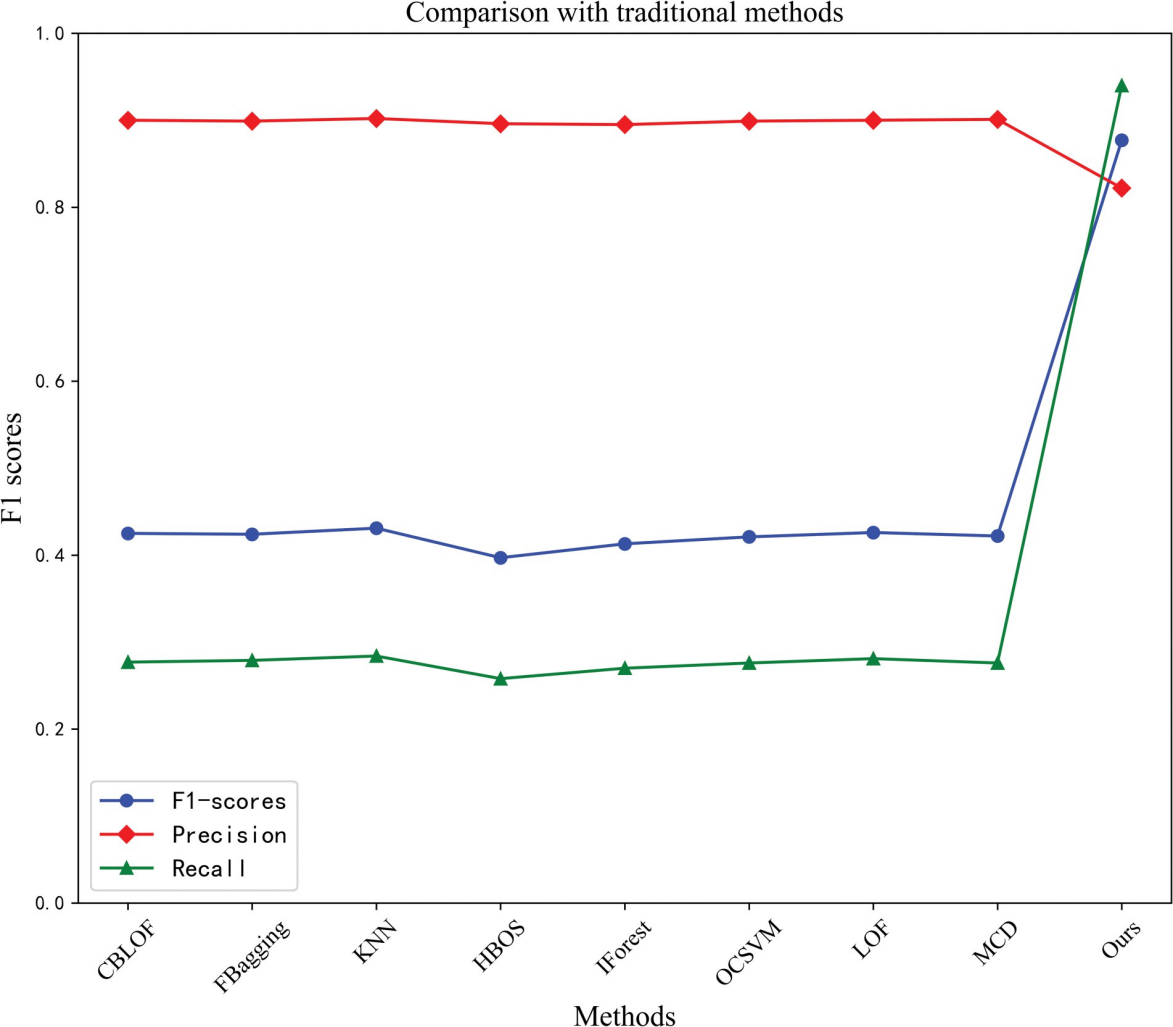
Comparison with traditional methods.

In the classical method model, KNN and MCD produce the highest precision, i.e., 0.902 and 0.901, respectively. The method in this paper is 0.822 in terms of precision, which is slightly lower than the above two models but much higher in terms of recall and F-score than the above two models, as shown in Fig 9.

**Fig 10:**
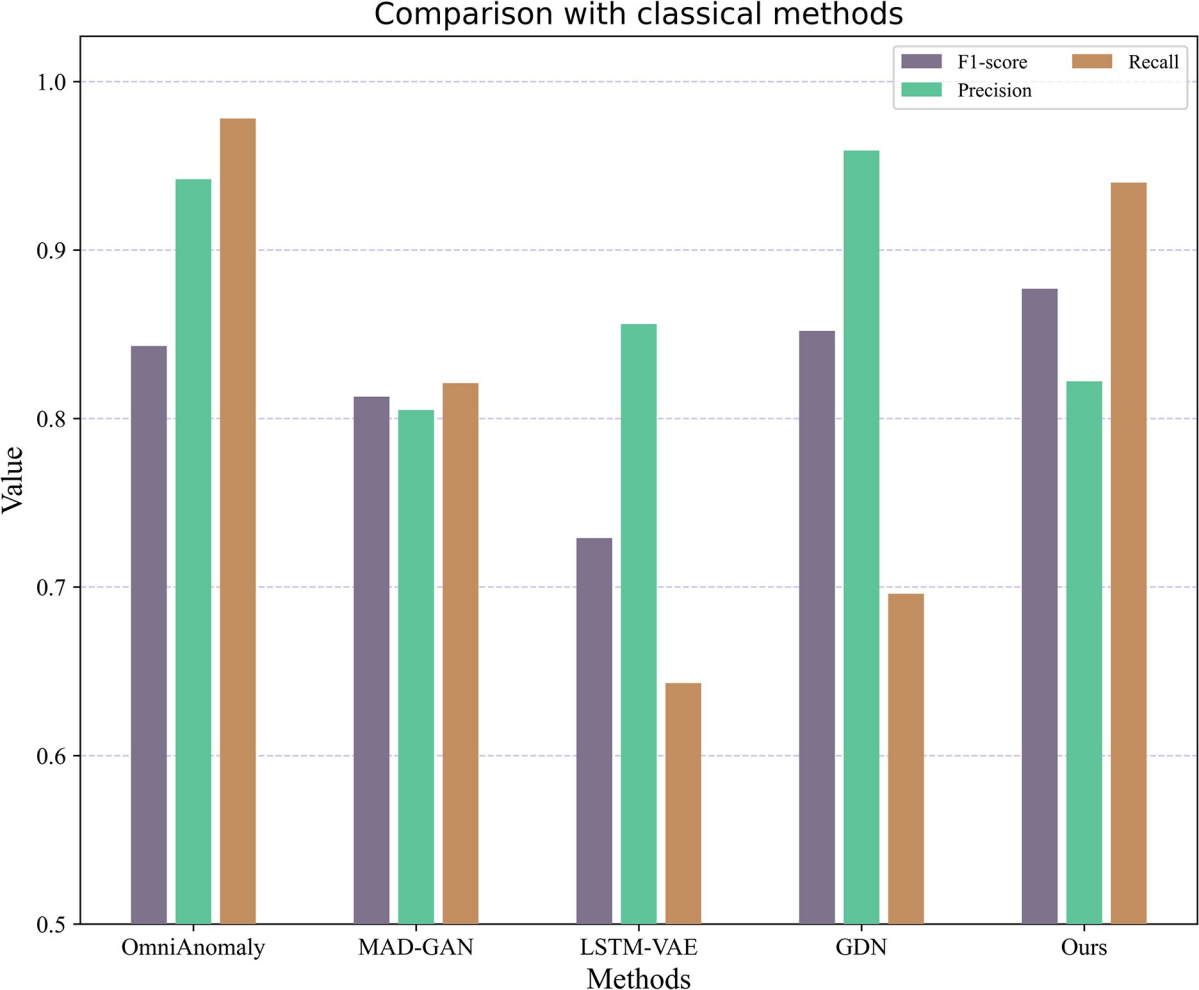
Comparison with advanced methods.

The figure shows that the F1-score of the OmniAnomaly model is only 0.843. The model cannot explicitly address the correlation between multiple features in the model, which is crucial to the success of multivariate time series anomaly detection and is the reason for the unsatisfactory F1-score. In this method, this problem is solved using a convolutional graph network, and the superiority of this method is verified in the experiments. The specific performance is shown in Fig 10.

The F1-scores, precision, and recall obtained by the method in this paper are 0.877, 0.822, and 0.940, respectively, where F1-scores and recall are higher than those of the MAD-GAN and LSTM-VAE models because the potential relationships between variables are ignored in the MAD-GAN and LSTM-VAE model methods, which only capture the intercorrelation of data in the time dimension without explicitly considering the relationship between different variables, which in turn leads to low model accuracy. Temporal information is also important for multivariate time series anomaly detection. In this paper, the LSTM is used to capture the long-term time-dependent relationships in the model, and the graph attention mechanism is used to calculate the attention scores between the relevant timestamps, which are designed to help achieve better performance.

### Parameter impact

**Fig 11:**
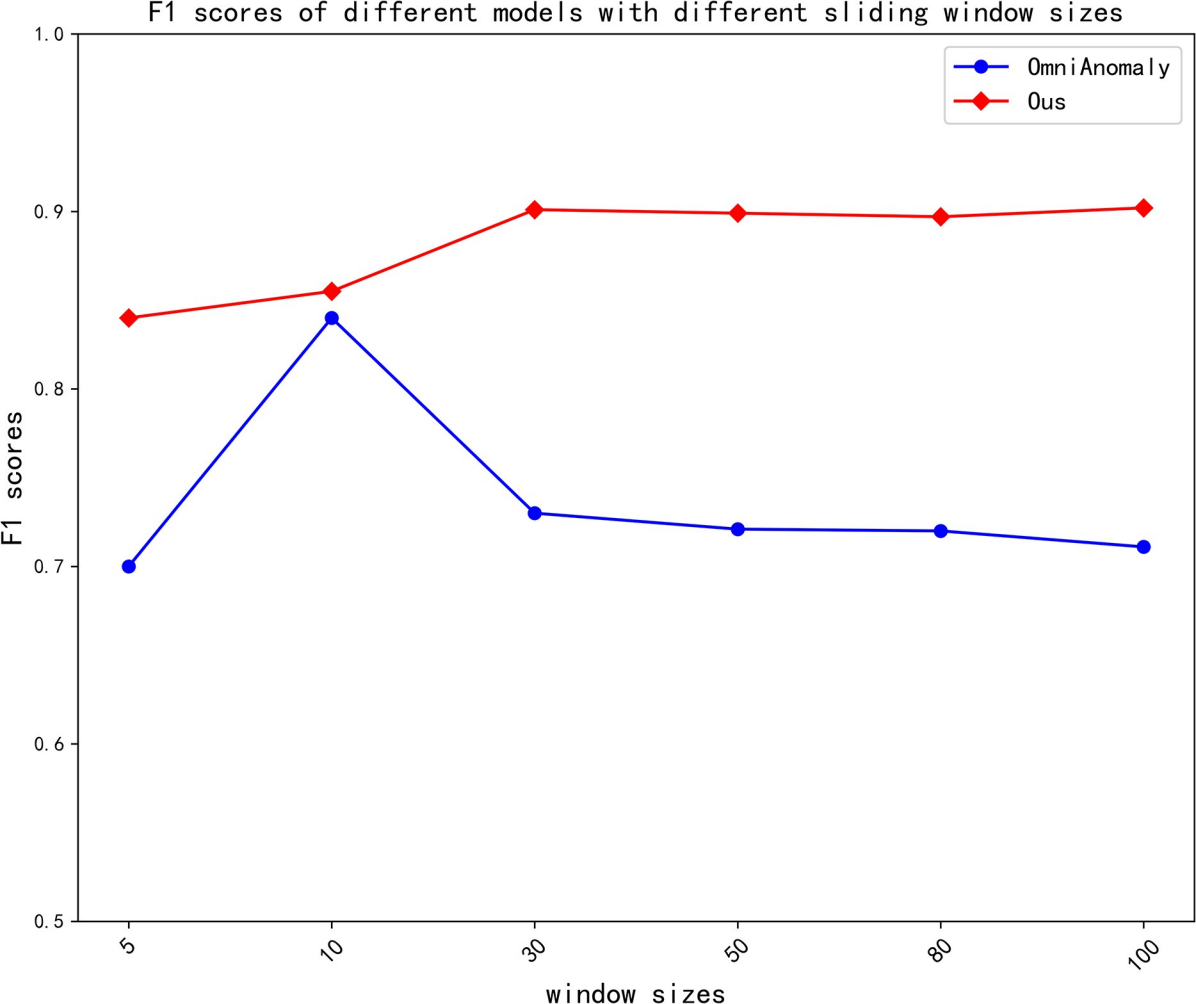
F1-scores of different models with different sliding window sizes.

Window size sensitivity: The F1-scores of this paper’s model were compared with its baseline approach under different sliding window sizes using the dataset. Since OmniAnomaly is more classical, the model of this paper was compared with it, with the same conditions except for the sliding window, and the results are shown in Fig 11. The smaller the size of the sliding window, the less content information is obtained, but because of the larger size of the sliding window, small anomalies in a short period of time are hidden in a long sequence, which leads to most baseline models being sensitive to the sliding window. Although OmniAnomaly is not sensitive to the size of the sliding window, its overall effect is not as good as the model in this paper. Based on this, the graph attention mechanism is used to make the model less sensitive to the sliding window size.

### Ablation experiments

To illustrate the necessity and effectiveness of the core components, an ablation study was conducted on the dataset to verify the time dependence of GRU learning, the dependence between multivariate time series features of GCN and GAT learning, and the role of the prediction module, which contributes to the improved results of the proposed model. On the dataset, the F1-scores of the model after removing each principal component were observed to measure its impact on the model.

First, name the model without the different components as follows:

1. w/o time: remove the GRU module and remove the time dependency.
2. w/o GAT: Remove the GAT module and remove the feature dependencies.
3. w/o Prediction: remove the prediction module and remove the prediction-based detection method

As shown in Table 4, when the GRU module is removed, the F1-score is reduced by about 2.6%, indicating that the model in this paper works well using the GRU module, taking into account the time series correlation. In the absence of the GAT module, the F1-score is reduced by 1.6% on average. This suggests that adding the GAT module provides a correlation between multiple feature variables and that it is useful to accurately capture this correlation for better anomaly detection. Removing the prediction-based detection method reduces the F1-score by an average of 3.1%. The prediction-based model predicts the actual value of the next timestamp in a deterministic manner, which is sensitive to capture the randomness of the time series and better for anomaly detection, thus allowing for a stable improvement in model performance.

**Table 4:** F1-score under the model without different components.

MODEL	F1-score
w/o time	0.854
w/o GAT	0.863
w/o Prediction	0.851

### Overhead analysis

The computational performance of the models in this paper is presented by comparing them with the classical methods OmniAnomaly and MAD-GAN baseline models. Table 5 shows the average training time in seconds for all models on the dataset.

**Table 5:** Average training time.

Methods	Average training time
OmniAnomaly	13.05
MAD-GAN	18.66
Ours	11.62

To ensure fairness in the experimental comparisons, we set the batch size and sequence length to be the same for all models. In general, models with LSTM or GRU require a longer training time. The MAD-GAN model uses a long-short-term memory recurrent neural network as the base model in the GAN framework to capture the temporal correlation of the time series distribution, resulting in a huge time overhead. As can be seen from Table 5, the model in this paper outperforms the OmniAnomaly, MAD-GAN model in terms of time overhead.

## Conclusions

In this paper, the following two key contributions are made. First, a multivariate time series anomaly detection method based on multivariate time series is proposed to consider multiple factors that lead to anomalies in building energy consumption. The anomaly detection framework is constructed using graph convolutional networks to extract feature correlations among multiple feature variables. Second, this paper introduces a graph attention mechanism to better achieve anomaly detection of building energy consumption by calculating the attention coefficients between different time series features and assigning greater attention weights to time series features with greater influence on energy consumption, which achieves better aggregation between global feature nodes. Using an open standard dataset, the performance of the method in this paper is compared with the baseline model for experiments and analysis, and the results show that the anomaly detection method has better detection accuracy than the baseline.

Future work is likely to come from two areas: first, addressing this problem of lack of annotation of datasets in the building domain, starting with the data pre-processing aspect to accurately annotate anomalous building energy datasets. Second, deepening the exploration of more feature-to-feature correlations and applying the models to more complex cases. Finally, more research should be conducted to facilitate anomaly detection techniques to reach their full potential in the building energy sector, such as the combination of Memristive LSTM networks and attention techniques in the paper [41] to enable importance assessment and interpretation of features, as well as the interpretation of how different features affect anomaly detection results.

## Supporting information

S1 Datasets (ZIP)
